# Inhibiting SIRT-2 by AK-7 restrains airway inflammation and oxidative damage promoting lung resurgence through NF-kB and MAP kinase signaling pathway

**DOI:** 10.3389/fimmu.2024.1404122

**Published:** 2024-06-24

**Authors:** Vandana Yadav, Vinita Pandey, Pratikkumar Gaglani, Atul Srivastava, Soni, Subhashini

**Affiliations:** ^1^ Department of Zoology, Mahila Mahavidyalaya, Banaras Hindu University, Varanasi, India; ^2^ Department of Biochemistry, Institute of Medical Sciences, Banaras Hindu University, Varanasi, India

**Keywords:** inflammation, innate immunity, MAP kinase, NF-kB, cytokines

## Abstract

**Introduction:**

Chronic obstructive pulmonary disease (COPD) is a major global cause of mortality with limited effective treatments. Sirtuins (SIRT) are histone deacetylases that are involved in the regulation of redox and inflammatory homeostasis. Hence, the present study aims to investigate the role of SIRT-2 in modulating inflammation in a murine model of COPD.

**Methods:**

COPD in mice was established by cigarette smoke (CS) exposure for 60 days, and AK-7 was used as the specific SIRT-2 inhibitor. AK-7 (100 µg/kg and 200 µg/kg body weight) was administered intranasally 1 h before CS exposure. Molecular docking was performed to analyze the binding affinity of different inflammatory proteins with AK-7.

**Results:**

Immune cell analysis showed a significantly increased number of macrophages (F4/80), neutrophils (Gr-1), and lymphocytes (CD4^+^, CD8^+^, and CD19^+^) in the COPD, group and their population was declined by AK-7 administration. Total reactive oxygen species, total inducible nitric oxide synthase, inflammatory mediators such as neutrophil elastase, C-reactive protein, histamine, and cytokines as IL4, IL-6, IL-17, and TNF-α were elevated in COPD and declined in the AK-7 group. However, IL-10 showed reverse results representing anti-inflammatory potency. AK-7 administration by inhibiting SIRT-2 decreased the expression of p-NF-κB, p-P38, p-Erk, and p-JNK and increased the expression of Nrf-2. Furthermore, AK-7 also declined the lung injury by inhibiting inflammation, parenchymal destruction, emphysema, collagen, club cells, and Kohn pores. AK-7 also showed good binding affinity with inflammatory proteins.

**Discussion:**

The current study reveals that SIRT-2 inhibition mitigates COPD severity and enhances pulmonary therapeutic interventions, suggesting AK-7 as a potential therapeutic molecule for COPD medication development.

## 1 Introduction

Chronic obstructive pulmonary disease (COPD) is a major and increasing global health problem associated with social and economic burdens (1). Currently, COPD is the fourth biggest cause of death worldwide, and it is anticipated to rank even higher by 2030 (2). COPD is an inflammatory condition leading to functional and structural alterations in airways, lung parenchyma, and pulmonary vasculature. The pathological condition is characterized by an airflow obstruction, parenchymal deterioration, and an emphysema-induced progression which leads to a permanent decrease in lung function (3). As per many reports, the main cause of COPD includes prolonged exposure to cigarette smoke (CS) which contains more than 4,700 toxic chemical components and 1,014 free radicals or oxidants (4). The toxic compounds of CS are the principal cause for the pathogenesis of COPD (5). Another key risk factor includes environmental and occupational exposure as silica dust, inorganic particles such as asbestos and cement, welding fumes, cadmium irritants, and alpha-1 antitrypsin deficiency, which may also play a crucial role in COPD etiology. Exposure to irritating gases such as nitrous oxide, sulphur dioxide, and chlorine has also been linked with the deterioration in lung function, whereas second-hand smoke (SHS) has been linked to an increased risk of COPD (6).

Numerous studies have shown that the pathophysiology and disease history of COPD are inextricably linked to oxidative stress, apoptosis, aging, and long-term immunological and inflammatory responses (7). However, CS exposure causes changes in structural and resident cells like endothelial, airway, alveolar epithelial cells, and fibroblasts which set off an inflammatory cascade that instinctively activates macrophages and airway epithelial cells releasing cytokines and chemokines (8). These released cytokines and chemokines further encourage the recruitment of other inflammatory cells (eosinophils, neutrophils, macrophages, and lymphocytes) at the inflamed site and initiate an innate immune response (9). Infiltration of neutrophils and macrophages is further accompanied by the release of several inflammatory mediators such as histamine, and C-reactive protein (CRP), neutrophil elastase (NE) (10). The severity and course of acute exacerbations of COPD reflect the capacity of the adaptive immune system in modulating pathogenesis with the participation of lymphocytes and dendritic cells. As a result of both innate and adaptive immune responses, the predominance of CD8^+^ and CD4^+^ cells is revealed in COPD. However, in the more severe cases, the presence of lymphoid follicles containing B lymphocytes and T cells (CD4^+^ and CD 8^+^) appears, resulting in a specific pattern of inflammation in the airways and parenchyma (11).

Exposure to CS changes the redox status within the cell, which also triggers the inflammatory responses promoting lung inflammation via the induction of the respiratory burst in phagocytic cells, control of intracellular signaling, chromatin remodeling (histone acetylation/deacetylation), and activation of redox-sensitive transcription factors like nuclear factor-kB (NF-kB) (12, 13). The latter is essential for the gene expression of pro-inflammatory mediators like interleukin (IL)-8, IL-6, and tumor necrosis factor-α (TNF-α), adhesion molecules, chemokines, growth factors, and enzyme which links CS exposure to altered cytokine production (14). However, NF-κB can also correlate with other transcriptional proteins such as histone acetyltransferase (HAT) and histone deacetylase (HDAC) (15).

Oxidants have been reported to elicit impact on mitogen-activated protein kinase (MAPK) signaling cascades, including extracellular signal-regulated kinases (ERKs), c-Jun N-terminal kinases (JNKs), and p38, which further exert substantial impacts on immunological responses in the airways (16). The most significant modulator of cellular transcriptional activity, including inflammatory responses, among MAPKs is ERK (17). Furthermore, many previous studies report the implication of nuclear factor erythroid 2-related factor (Nrf-2) induction by MAPK signaling in response to diverse stimuli, including oxidative stress. It has been reported that Nrf-2 phosphorylation at several serine and threonine residues is initiated by MAPKs. *In vivo* phosphorylation of Nrf-2 at multiple sites by MAP kinases has a limited contribution in modulating the Nrf-2-dependent antioxidant response (18).

Epigenetic processes, including DNA methylation, post-translational modifications of histones (histone methylation, acetylation, phosphorylation, ubiquitination, and sumoylation), and non-coding RNAs may impact reactivity to CS and oxidants in the immune cells of lungs in COPD pathology and modulates chromatin structure and gene expression (19). Collectively, studies have emerged with evidence showing that epigenetic mechanisms impact on phenotypic changes in lung disease. Understanding the epigenetic mechanism can help to explore the pathogenesis and identify novel targets for developing new therapies for COPD patients. Moreover, recent publications have revealed the ability of various approaches, such as sirtuins (SIRT), to modulate HDAC2 activity, representing a therapeutic effect in COPD (20).

SIRT are class III HDAC that are NAD-dependent and control epigenetic modifications to affect both normal and pathological functions in lung disease (21). Due to their potential to influence inflammatory and various cellular processes associated to redox, antioxidant signaling SIRT have also been discovered as important therapeutic targets for the treatment of lung disorders (22). In order to control the expression of genes, SIRT also deacetylate a variety of transcription factors and enzymes, including p53, NF-kB (a key player in a variety of inflammatory networks which regulates cytokine release in airway disease), and DNA-dependent protein kinase (23). These are also reported to be linked with the emergence of airway inflammation in bronchial asthma, COPD, acute respiratory distress syndrome, and lung cancer (24). It is assumed that SIRTs (1, 2, 6, and 7) may be crucial for controlling inflammatory responses (25). SIRT-1 plays an important role in regulating the inflammatory pathogenesis of COPD by negatively regulating NF-κB, reducing FOXO3 acetylation, inhibiting STAT3 activation, and preventing airway remodeling. SIRT-2 deacetylates p65, which regulates the expression of NF-kB-dependent genes. However, studies also reflect that SIRT-2 mutations are key COPD risk factors as they promote chronic systemic inflammation and oxidative stress. A preclinical study using SIRT-2 observes that elevation in asthmatic lungs exerts pro-inflammatory effects (26). SIRT-2 has been extensively examined for its involvement in influencing senescence, myelin formation, autophagy, and inflammation. These has been linked with numerous neuroinflammatory pathologies as it contains deacetylation characteristics that affect immune homeostasis (27). SIRT-3, SIRT-4, and SIRT-5 are mitochondrial proteins that are strongly connected to mitochondrial function and play a key role in the development of COPD-associated mitochondrial dysfunction. SIRT-6 controls oxidative stress and also reduces TGF-β-induced senescence via p21 proteasomal degradation and IL-1 release to prevent fibrosis. SIRT-7 regulates DNA damage repair, the cell cycle, and aging, implying that it is also involved in the pathophysiology of COPD (28).

Among *in silico* studies, molecular docking is a popular computational approach for studying molecular recognition, with the goal of predicting the binding mechanism and affinity of a complex produced by two or more constituent molecules with known structures. Protein–ligand docking is an essential form of molecular docking representing its therapeutic uses in contemporary structure-based drug discovery (29). Molecular recognitions, including enzyme–substrate, drug–protein, drug–nucleic acid, protein–nucleic acid, and protein–protein interactions, play important roles in many biological processes such as signal transduction, cell regulation, and other macromolecular assemblies. Therefore, determination of the binding mode and affinity between the constituent molecules in molecular recognition is crucial to understand the interaction mechanisms and design therapeutic interventions (30).

A previous study from our lab based on similar experiments conducted in COPD mice model using AK-7 as SIRT-2 inhibitor has proven it to regulate oxidative stress (oxidant and antioxidants) through regulating Nrf-2 (31). Based on the previous findings, it is hypothesized that SIRT-2 may further play an important role in the pathophysiology of COPD by modulating redox-sensitive transcription factors such as NF-kB, inflammatory, and immunological parameters. Hence, the present study has been designed to investigate the role of SIRT-2 in COPD by its pharmacological inhibition by AK-7 and to further explore its role in regulating inflammatory mediators and pathways in COPD pathogenesis.

## 2 Materials and methods

### 2.1 Chemicals

Capstan Wills Navy cigarettes, manufactured and distributed by ITC Limited in India, AK-7 [3-(1-azepanylsulfonyl)-N- (3-bromophenyl)) benzamide], 2′,7′-dichlorofluorescein diacetate (DCFDA), and glutaraldehyde were purchased from Sigma-Aldrich (USA). Sodium dodecyl sulphate (SDS), opthaldehyde (OPT), O-phenylene-diamine-dihydrochloride (OPD), histamine, and paraformaldehyde were obtained from Sisco Research Laboratories Pvt. Ltd. (SRL), India. The cytokine ELISA kits (IL-5, 6, 10, and 17) and cell surface markers against neutrophil (Gr-1), T-naïve cells (CD3^+^), T-cytotoxic (CD8^+^), T-helper (CD4^+^), macrophages (F4/80^+^), and B-cells (CD19^+^) were procured from Biolegend (USA). Neutrophil elastase (NE) chromogenic substrate (MeOSuc-AAPV-pNA) and antibodies against phospho-ERK, phospho-JNK, phospho-MAPK, and phospho-NF-kB were bought from Santa Cruz. Antibody against β-actin and goat anti-mouse IgG antibody (HRP-conjugated) were purchased from Genscript (cat. no.: A00160). Trizol (cat. no. 9108) was bought from Takara. Revert Aid first-strand cDNA synthesis kit (cat. no. K1622) and maxima SYBR Green/ROX qPCR mastermix kit (cat. no. K0221) were purchased from Thermos Fisher Scientific. Primers against specific genes were bought from Eurofins Genomics India Pvt. Ltd.

### 2.2 Experimental animals

Balb/c mice were used as experimental animals to develop COPD. Mice weighing 18 to 22 gm and aged 6 to 8 weeks were procured from the Central Drug Research Institute, Lucknow, India.

They were housed in a tidy animal housing, with autoclaved husk bedding in polypropylene cages. All mice were acclimatized for 1 week under conventional and standard animal housing facilities (temperature, 25°C ± 3°C; humidity, 60%–70%) and subjected on a 12-h diurnal cycle before the experiment. All experimental mice were maintained under pathogen-free circumstances with access to sufficient feed and ad libitum water. The experiment protocols and animal care were approved and conducted in compliance with the guidelines of the Institutional Animal Ethical Committee (IAEC), Banaras Hindu University, Varanasi, India, as per the letter no. BHU/DoZ/IAEC/2021–2022/017 dated 15/02/2022.

### 2.3 Experimental grouping of animals

**Figure 1:**
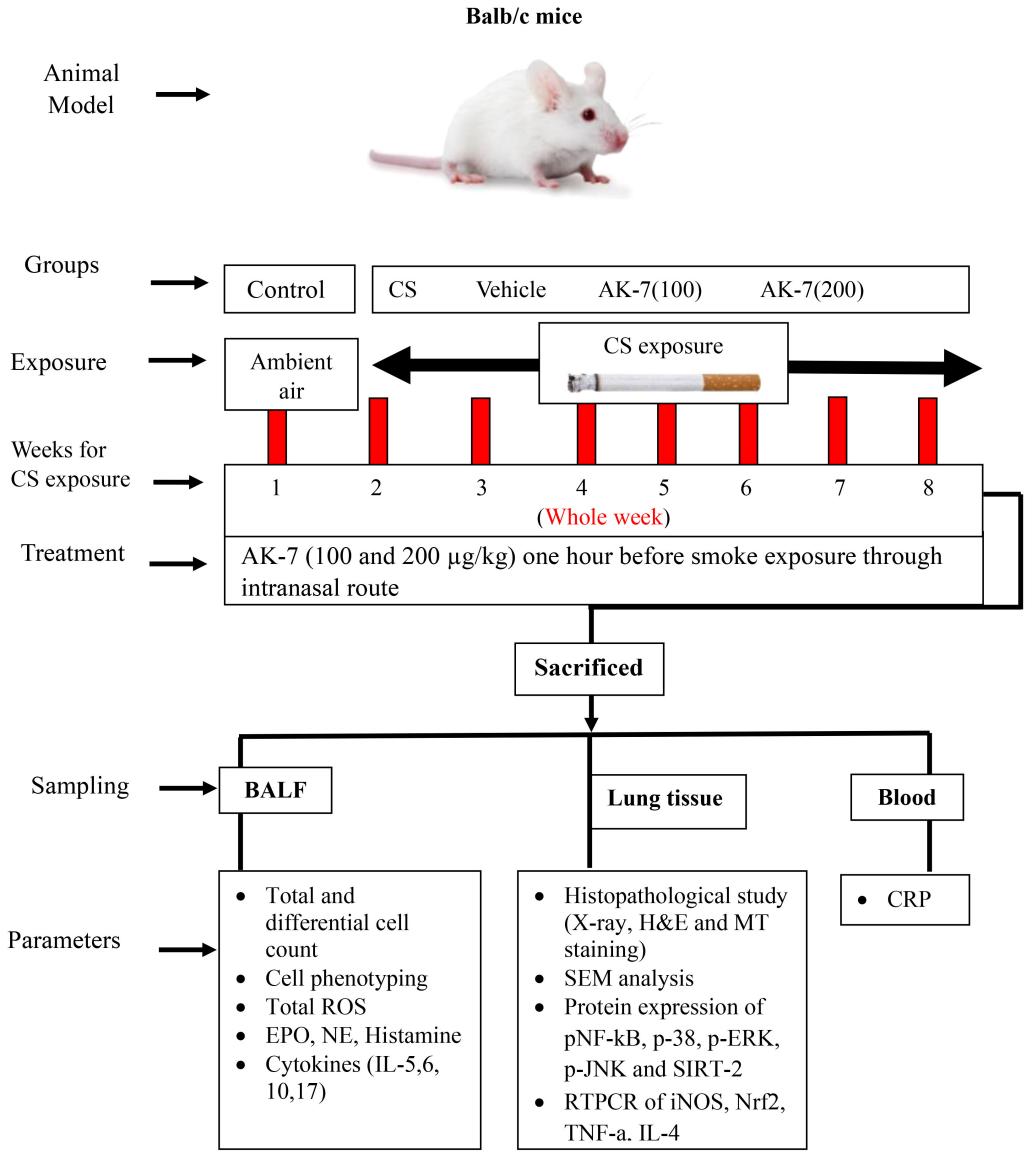
Diagrammatic representation of experiment protocol, sampling, and parameters studied.

A total of 40 mice were randomly divided into five groups (eight mice/group) as shown in **Table 1**. Group I consisted of control mice exposed to ambient air, group II comprised of COPD mice exposed to cigarette smoke (CS), group III consisted of mice administered with vehicle (DMSO diluted in PBS, the AK-7 solvent), group IV consisted of mice administered with AK-7 (100 µg/kg bw), and group V consisted of mice administered with AK-7 (200 µg/kg bw) (**Figure 1**).

**Table 1:** Grouping of animals.

Sr. no.	Groups	Inducer	Treatment (intranasal route 1 h before the cigarette smoke exposure)
1	Control	Exposed to ambient environmental air	–
2	COPD	Exposed to 1 cigarette smoke/day for 2 months	–
3	Vehicle	Exposed to 1 cigarette smoke/day for 2 months	DMSO diluted in PBS
4	AK-7(100 µg/kg)	Exposed to 1 cigarette smoke/day for 2 months	AK-7 (100µg/kg bw)
5	AK-7(200 µg/kg)	Exposed to 1 cigarette smoke/day for 2 months	AK-7 (200µg/kg bw)

### 2.4 Development of CS-induced COPD in mice

CS was used to induce COPD by placing the mice in a closed whole-body CS inhalation chamber, with a fan to circulate oxygen throughout the chamber. The chamber also has a smoke-generating room connected in series to an inhalation chamber by silicone tubes. The experimental mice were exposed to one non-filtered CS every day for 60 days to establish a COPD condition (32, 33). Except for the control mice, the mice of all other groups (II, III, IV, and V) were exposed with CS for the development of COPD. Control mice was exposed to ambient air in the same manner as CS.

### 2.5 Administration of AK-7

AK-7 (dissolved in 1% DMSO mixed in PBS) was delivered through intranasal route (i.n.) at a dose of 100 μg/kg bw (group IV) and 200 μg/kg bw (group V) 1 h before CS exposure (34). Group III received i.n. DMSO and PBS (AK-7 solvent) 1 h before smoke exposure.

### 2.6 Body weight and X-ray analysis of lungs

The body weight (bw) of mice was observed at the beginning of the experiment and further monitored weekly. At the end of the experiment, the change in bw was calculated. The lungs of the animals were X-rayed 24 h before sacrificing the experimental animal to access changes in pulmonary anatomy. The mice were anesthetized and placed in ventral position to capture an image of the chest. The X-ray source was run at 35 kVp (source power: 17 W) for image collection. The exposure duration for each animal was 5 s (35).

### 2.7 Collection of bronchoalveolar lavage fluid, blood, and lungs

The animals were sacrificed on the 61st day, 24 h following the final CS exposure. Blood serum was collected by retro-orbital bleeding and used to analyze the CRP and histamine content. Bronchoalveolar lavage fluid (BALF) was obtained by tracheal cannulation and washing the lung lumen. The airway lumen was infused and rinsed three times with 1 mL chilled phosphate buffer saline (PBS). The total retrieved supernatant was centrifuged for 10 min at 4°C and 3,000 rpm. Briefly, 200 µL of PBS was used to resuspend the BALF pellets. ROS and immune cell markers were analyzed from BALF pellets, and cytokine profiling, histamine, EPO, and NE assays were performed in BALF supernatant. Lung tissue was used for histological studies, protein, and mRNA expression.

### 2.8 Total and differential cell count

Briefly, 10 µl of the BALF pellet suspension was stained with trypan blue, and the total number of inflammatory cells was counted in a hemocytometer (36). Additionally, 50 µL of the pellet suspension was added to gelatin-coated slides and cytocentrifuged in a cytospin machine for 10 min at, 2000 rpm at 4°C. The slides were fixed in methanol and stained with Giemsa stain. According to their nuclear morphology, the cells were recognized and counted in a total of 100 in different areas.

### 2.9 Flow cytometry analysis to analyze inflammatory cell recruitment

The collected BALF pellet was dissolved in 100 μL FACS buffer (2% fetal bovine serum and 0.2% sodium azide in PBS) and treated with lysis buffer (ammonium chloride) to burst RBCs. Furthermore, ∼6 × 10^4^ cells were counted and further processed with a different marker for cell phenotyping. Cell surface marker expression on T lymphocytes, macrophages, granulocytes, neutrophils, and B lymphocytes were examined using particular antibodies conjugated with fluorescent dyes, such as PE-anti-CD4 (Th-2), anti-F4/80 (macrophages), anti-CD19 (B-cells), FITC-anti-CD8 (Th1), anti-CD3 (naive T-cells), and APC anti-Gr-1 (neutrophils) (Biolegend, USA), as per the manufacturers instruction. The samples were incubated with their respective antibody for 15 min at room temperature in the dark. Each individual cell population was recognized and counted based on the fluorescence color (37).

### 2.10 Reactive oxygen species measurement

The previously published methodology was used to measure the intracellular reactive oxygen species (ROS) level in BALF pellet cells (38). The BALF pellet cells were washed in cold PBS, and their overall viability was quantified using trypan blue dye. Moreover, 1 × 10^5^ cells in 100 µL PBS were incubated with 100 µL of 10 μM of DCFDA at 37°C for 45 min in the dark, and the cells were then placed on ice for immediate detection by flow cytometry (Beckman Coulter, CytoFLEX LX). Data was acquired and analyzed using the CELL Quest program.

### 2.11 Eosinophil peroxidase activity

Estimation of eosinophil peroxidase (EPO) was performed by using a previously described protocol (39). The eosinophil peroxidase levels in BALF were determined by using 100 µL substrate containing 0.1 mM O-phenylenediamine dihydrochloride, 0.1% Triton-X-100, 100 mM H_2_O_2_, and 0.05 M Tris HCl in DW. Moreover, 100 µL of substrate was incubated with 100 µL BALF in a microplate and incubated for 30 min at 37°C. The reaction was stopped by adding 50 µL of 4 M sulfuric acid, and absorbance was taken at 490 nm. The activity of the enzyme was expressed as absorbance at 490 nm.

### 2.12 Neutrophil elastase

Neutrophil elastase (NE) assay was performed by using an established protocol with slight modifications (40). MeOSuc-AAPV-pNA, a synthesized peptide, was used as chromogenic substrate for binding with NE. In brief, 0.1 M HEPES buffer was prepared by adding 0.5 M NaCl. The reaction mixture was prepared by adding 73.5 µL of 0.5 mM substrate with 126.5 µL HEPES buffer and 50 µL BALF. The reaction mixture was incubated at 37°C for 48 h. NE was analyzed by observing the absorbance at 405 nm.

### 2.13 C-reactive protein

C-reactive protein (CRP) was estimated as per the described protocol by nephelometry method (41). Serum was mixed with 20% Tris-calcium buffer (pH, 7.5) and incubated at 37°C for 12 min. Furthermore, CRP–phospholipid complexes were measured by using a nephelometer. The results were calibrated using CRP as standard and expressed as mg/dL.

### 2.14 Estimation of histamine

Histamine estimation was performed by an earlier established protocol (42). Histamine stock solution was prepared in 0.1 M HCl at -20°C. The released histamine was calculated in a flat-bottomed black plate in the dark. Briefly, 50 µL BALF was diluted with 50 µL 0.1 M HCl (1:1), and 36 µL of 1 M NaOH was added. Later, 9 µL of OPT (10 mg/mL) dissolved in 1% methanol was added to the reaction mixture and incubated in the dark. After 20 min, the reaction was stopped by adding 18 µL of 3 M HCl. Fluorescence intensities were read at 360-nm excitation and 450-nm emission filters. The results were expressed as µg/mL.

### 2.15 Estimation of cytokines

Commercially available kits (Biolegend, USA) were used to conduct an enzyme-linked immunosorbent assay (ELISA) to measure the levels of different cytokines such as IL-5, IL-6, IL-10, and IL-17 in the BALF supernatant as per the manufacturer’s instructions. The results were expressed in pg/mL.

### 2.16 Histopathological alterations in lung tissues

Lung tissue fixed in 10% neutral buffer formalin (NBF) was dehydrated in ascending grades of ethanol, cleared in xylene, and embedded in paraffin wax. Then, 5-μm-thin sections were cut using microtome (Leica); staining was performed with hematoxylin and eosin (H&E) for the analysis of architectural changes and inflammatory cell infiltration, and Masson’s trichrome (MT) staining was used to elucidate airspace remodeling (collagen deposition) in the lungs. The mean linear intercept (Lm) was determined to quantify airway enlargement and alveolar destruction. In histopathological slides, inflammation and collagen content were scored and assessed semi-quantitatively on a scale ranging from grade 0 to grade 4 to estimate the changes as follows: grade 0—no inflammation and collagen; grade 1—inflammation in the lungs in a single layer, few collagen in the alveolar region; grade 2—inflammation in the lungs in two to three layers, few collagen in the alveolar region; grade 3—inflammation in the lungs in several layers, collagen in alveolar, peribronchiolar, and perivascular regions; grade 4—inflammation in the lungs in several layers in full alveolar region, thick collagen band in the alveolar, peribronchiolar, and perivascular regions (43).

### 2.17 Scanning electron microscope (SEM) analysis of the lungs

The lungs were excised, sliced into 2-mm size, and fixed in 10% buffered formaldehyde and glutaraldehyde (2.5%) solution with 1 M sodium cacodylate. Furthermore, tissue sections were dehydrated through ascending grades of acetone and dried using a desiccator for 48 h. Tissue was attached to the specimen holders and coated with conductive gold film of roughly 30 nm in thickness. The tissues were further scanned and imaged with a Zeiss Sigma 300 SEM (FEG Oberkochen, Germany) in the secondary electron (SE) and backscattered electron (BSE) modes (44).

### 2.18 Western blotting for the expression of proteins NF-kB, p-p38, p-ERK, p-JNK, and SIRT-2

Lung proteins were prepared in a homogenate buffer (20 mM Tris, pH 7.5, and 150 mM sodium chloride) containing a protease inhibitor cocktail (1 mM sodium orthovanadate, 1 mM PMSF, and 1 μM Aprotinin). After determining the protein concentration by the Lowry method, 120 μg of proteins was separated on 12% SDS-PAGE gels and then transferred onto nitrocellulose membranes (45). The membranes were blocked with 5% non-fat skimmed milk in Tris-buffered saline with 0.01% T-20 (Tween 20) for 2 h, followed by incubation with primary antibodies to β-actin (1:1,000 Santa Cruz) antibody overnight at 4°C. After washing the membranes with TBST for 15 min, the membranes were probed with horseradish peroxidase (HRP)-conjugated secondary antibody (anti-mouse, 1:10,000, Real Gene) and were visualized by ECL reagent (BioRad). Protein bands were analyzed by densitometry, and band intensities were determined using Image J software.

### 2.19 RNA extraction and reverse transcription-quantitative polymerase chain reaction analysis for iNOS, Nrf-2, TNF-α, and IL-4

Total RNA was extracted from the lungs using Trizol reagent as per the established protocol and assessed for quantification through ultraviolet absorption at 260/280 nm (46). Complementary DNA (cDNA) was synthesized using Revert Aid cDNA synthesis kit. The expressions of different genes were assessed by reverse transcription-quantitative polymerase chain reaction (RT-qPCR) using specific forward and reverse primers along with SYBR Green mastermix kit. 2^−ΔΔCq^ method was used to determine the relative mRNA levels by using GAPDH as an internal control. The sequences of the forward and reverse primers used are listed in **Table 2**.

**Table 2:** List of primer sequences.

Primer	Forward primer	Reverse primer
GAPDH	GCCAAAAGGGTCATC	GTAGAGGCAGGGATGC
TNF-α	GCCTCTTCTCATTCCG	CTGATGAGAGGGAGGAT
Nrf-2	CTGAACTCCTGGACA	CGGTGGGTCTCCGTAAG
IL-4	ATCATCGGCATTTTGC	ACCTTGGAAGCCCTACA
iNOS	TTGGAGCGAGTTGTGG	GTAGGTGAGGGCTTGGA

### 2.20 Molecular docking analysis

Molecular docking of different proteins with the ligand AK-7 was performed to determine their binding affinity. The structure of proteins was taken from Protein Data Bank (PDB) (https://www.rcsb.org/), and ligand structure was retrieved from PubChem database (https://pubchem.ncbi.nlm.nih.gov/). The proteins were prepared for docking by removing the water molecules and other hetero atoms and further adding polar hydrogen atoms, missing atoms, and Kollman charges to the residues, with the help of Discovery Studio (https://discover.3ds.com/). Ligand preparation was done by minimization of energy by Autodock Vina. Furthermore, docking was performed using Autodock Vina 4.2 by taking exhaustiveness of 100 runs. The results was analyzed as per the binding affinity of different proteins with the ligand (AK-7).

### 2.21 Statistical analysis

Experimental data were expressed as mean ± SEM (*n* = 8/group). The experiments were repeated thrice for authentication of the results obtained. The results obtained from each experimental group were analyzed by applying Student’s *t*-test and one-way ANOVA followed by Tukey’s test. The statistical analysis was carried out using Statistical Package (SPSS 26 for Windows), and *p*-values <0.05 were considered significant.

## 3 Results

### 3.1 AK-7 restores bw and inhibits CS-induced inflammatory cell recruitment (cell phenotyping)

**Figure 2:**
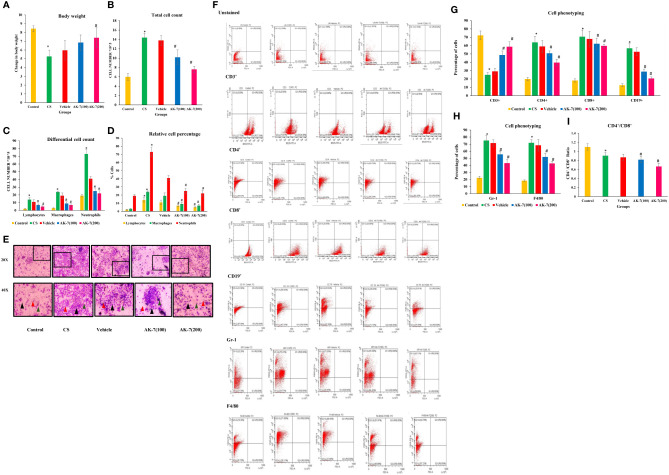
**(A)** Body weight, **(B)** total cell count, **(C)** differential cell count in BALF, **(D)** relative percentage of different types of cells, **(E)** Giemsa-stained cells of BALF at ×20 and ×40 magnification, **(F)** analysis of CD3^+^, CD4^+^, CD8^+^, CD19^+^, Gr-1, and F4/80, **(G)** cell phenotyping: graphical representation of lymphocytes (CD3^+^, CD4^+^, CD8^+^, and CD19^+^) in BALF, **(H)** cell phenotyping of Gr-1 and F4/80, and **(I)** ratio of CD4^+^/CD8^+^ cells. Body weight was declined in the CS group compared with the control, and the AK-7(200) group has significantly recovered body weight after 60 days of treatment when compared to the CS group. The CS group had higher counts of total cells which included macrophages, lymphocytes, and neutrophils in the BALF compared with the control. AK-7 administration reduced the number of cells along with macrophages, neutrophils, and lymphocytes compared with the CS group in a dose-dependent manner. Furthermore, FACS analysis showed a significantly higher population of T cells, B cells, neutrophils, and macrophages in the CS-exposed group compared with the control group. The AK-7 group shows a significant reduction in their population compared with the CS group. The CD4^+^/CD8^+^ ratio in the CS group showed a lower value compared with the control, which represents recruitment of more T-cytotoxic cells compared with T-helper cells. In spite of the decline in population percentage, the AK-7 group simultaneously maintains this ratio. The number of different cells in the vehicle group was higher than the control and lower than the AK-7 group. Number of mice per group (*n* = 8). Results were represented as mean ± SEM (*p < 0.05 control vs. CS group and #*p* < 0.05 CS vs. AK-7 group). Arrow representation: black, neutrophils; red, macrophages; green, lymphocytes.

The bw of experimental animals recorded from the beginning of the experiment followed by weekly observation represented a significant decline in the CS group compared with the control. Furthermore, AK-7(200) administration resulted in considerable recovery compared with the CS exposed group (**Figure 2A**). Any foreign stimuli entering the airways activates the immune system, causing the recruitment of different immune cells. In the present study, to elicit inflammation resulting from inflammatory cells, total cell count, differential cell count, and immunophenotyping were performed. The total numbers of cells in BALF of the CS group displayed greater infiltration of immune cells compared with the control, and the number of cells was dramatically reduced in the AK-7 group compared with the CS-induced COPD group, showing inhibition in recruitment of inflammatory cells by AK-7 administration (**Figure 2B**). Furthermore, BALF cells localized on slides for cytospin to analyze the cellular profile revealed a significantly higher number of cells per unit area in the CS group and the vehicle group, including neutrophils, lymphocytes, and macrophages (**Figures 2C–E**). AK-7 administration in mice significantly inhibited the lymphocyte, macrophage, and neutrophil recruitment in the airways. Furthermore, these results were confirmed through FACS analysis by staining the BALF cells with different cell markers (**Figure 2F**). The study revealed that Gr-1 (neutrophils) and F4/80 (macrophages) cell populations increased in COPD (75.23% and 71.89%) compared with the control (22.26% and 18.30%), and decreased in AK-7 groups where neutrophils represent 55.58% [AK-7(100)] and 43.71% [AK-7(200)], and macrophages showed 51.91% [AK-7(100)] and 42.78% [AK-7(200)] (**Figure 2H**). Abundance of neutrophils was found in all groups compared with macrophages and other cell variants.

CD3^+^ cells, which represent naive T-cells, was decreased in the CS exposed group (24.99%) compared with the control (72.30%), and an increased population of 48.76% was observed in AK-7(100) and 58.76% in AK-7(200). The population of different lymphocytes as CD4^+^, CD8^+^, and CD19^+^ was increased in the CS-induced COPD group (63.85%, 70.68%, and 56.82%, respectively) compared with the control (19.98%, 18.22%, and 12.60%). The population declined the in AK-7(100) group (50.76%, 62.23%, and 28.82%) and the AK-7(200) group (39.70%, 59.65%, and 20.61%) in a dose-dependent manner (**Figure 2G**).

The ratio of CD4^+^/CD8^+^ cells was found to be less than 1 in the CS-exposed group, representing the recruitment of more Tc cells compared with Th cells (**Figure 2I**). In spite of the decline in population percentage, the AK-7 group simultaneously maintained the ratio.

### 3.2 Effect of AK-7 on total ROS

**Figure 3:**
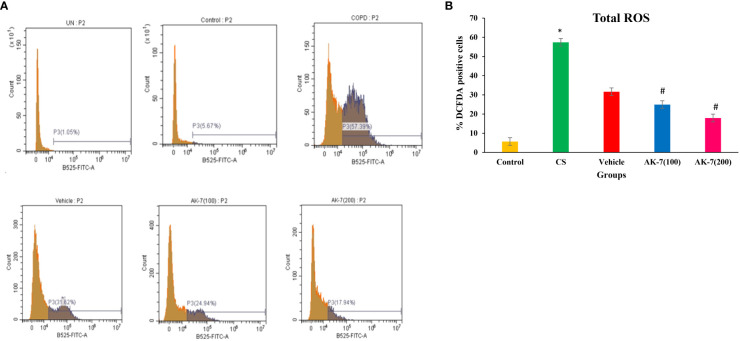
**(A)** Determination of the effect of AK-7 on total ROS in BALF. **(B)** Graphical representation of total ROS in different groups. Total ROS was significantly increased in CS-exposed and vehicle groups compared with the control group and significantly decreased in the AK-7 group compared with the CS group. Number of mice per group (*n* = 8). Results were presented as mean ± SEM (*p < 0.05 control vs. CS group and #*p* < 0.05 CS vs. AK-7 group).

Total ROS was detected in BALF through flow cytometry using fluorescent dye DCFDA (**Figure 3A**). The CS-induced COPD (57.39%) and vehicle (31.62%) groups exhibited a significantly higher fluorescence compared with the control group (5.67%), directing the presence of high intracellular ROS. AK-7 treatment significantly reduced the total ROS as 24.94% in AK-7(100) and 17.94% in AK-7(200) compared with the CS group (**Figure 3B**).

### 3.3 Effect of AK-7 on inflammatory markers to the lungs

**Figure 4:**
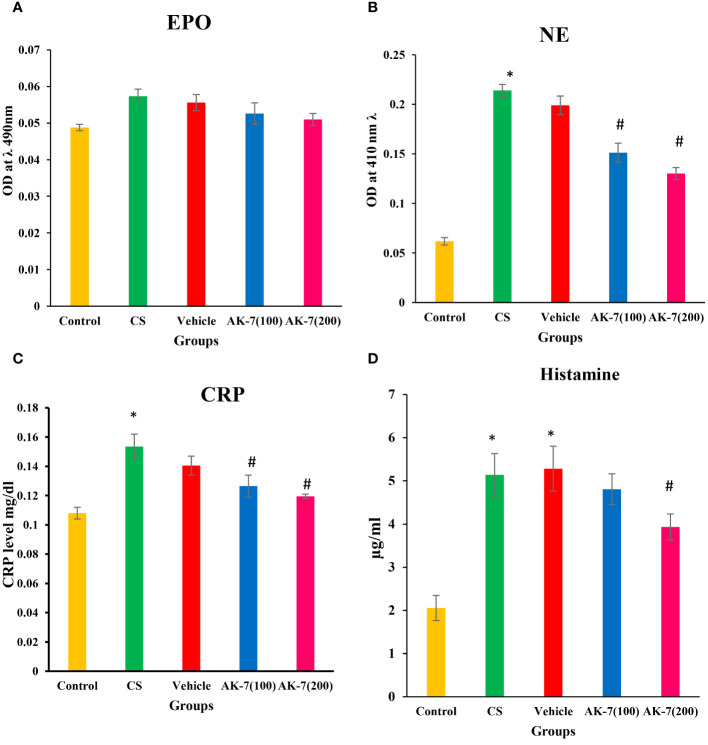
Determination of inflammatory markers **(A)** EPO, **(B)** NE, **(C)** CRP, and **(D)** histamine. The EPO level shows non-significant difference among different groups. NE, CRP, and histamine were significantly higher in the CS group compared with the control. The AK-7 group shows a significant reduction in NE and CRP compared with the CS group. Histamine was significantly decreased in AK-7(200) but not in AK-7(100). The NE, CRP, and histamine level in the vehicle group was found similar to that of the CS-exposed group, indicating the non-protective role of solvent in CS exposure. Number of mice per group (*n* = 8). Results were presented as mean ± SEM (*p < 0.05 control vs. CS group and #*p* < 0.05 CS vs. AK-7 group).

Recruitment and activation of eosinophils and neutrophils were assessed by the activity of EPO and NE, which represent their activating markers, respectively. A non-significant difference among different groups was observed in EPO activity, indicating non-effectiveness of CS on eosinophilic recruitment (**Figure 4A**). However, NE activity was significantly increased in the CS group compared with the control and was significantly reduced in AK-7 treatment groups in a dose-dependent manner, reflecting that SIRT-2 inhibition reduces neutrophilic infiltration in the lungs (**Figure 4B**). The vehicle group shows a result similar to the CS-induced COPD group.

The levels of CRP (**Figure 4C**) and histamine (**Figure 4D**), as a marker of inflammation, were assessed in serum and BALF, respectively. CRP and histamine were significantly higher in the CS group compared with the control. Both markers represented a significant reduction in AK-7 group compared with the CS group. However, the histamine level was significantly decreased in the AK-7(200) group only when compared to the CS-exposed group. The vehicle group shows a similarity with the COPD group in both CRP and level of histamine.

### 3.4 AK-7 modulated cytokine levels, such as IL-4, IL-5, IL-6, IL-10, IL-17, and TNF-α

**Figure 5:**
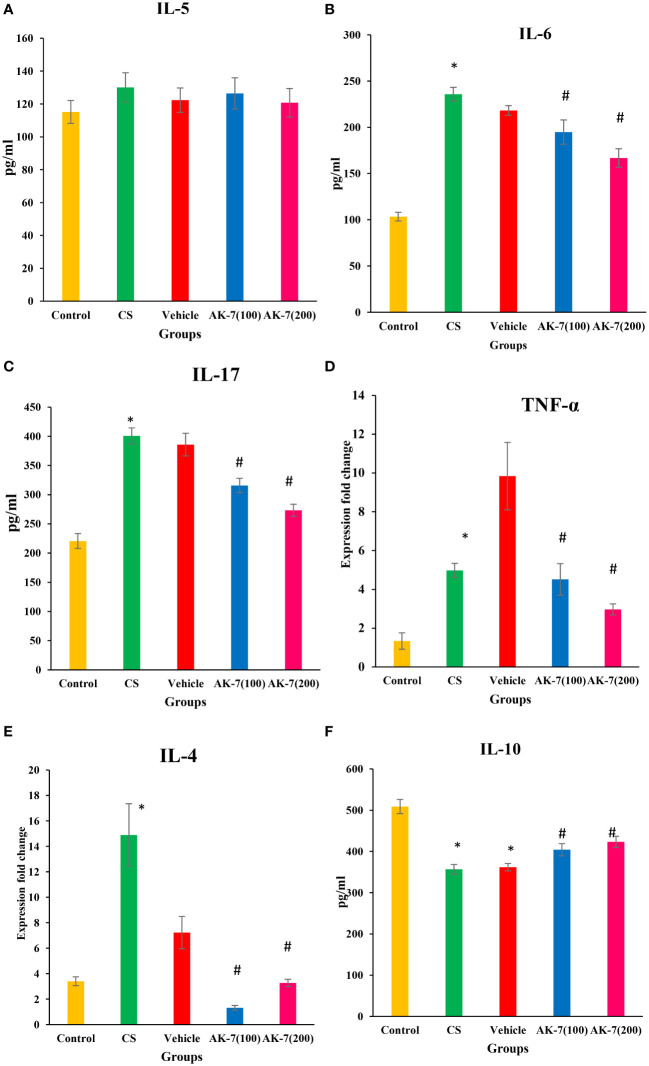
Effect of CS exposure and AK-7 on the level of different cytokines: **(A)** IL-5, **(B)** IL-6, **(C)** IL-17, **(D)** TNF-α, **(E)** IL-4, and **(F)** IL-10. IL-5 shows no significant difference among groups. The level of IL-4, IL-6, IL-17, and TNF-α was significantly increased in the CS group compared with the control group, while AK-7 downregulated the level of these cytokines compared with the CS-exposed group. The level of cytokines in the vehicle group was found near that of the CS group. IL-10 was significantly decreased in the CS-exposed and vehicle group compared with the control group and was upregulated in the AK-7 group compared with the CS group. Number of mice per group (*n* = 8). Results are presented as means ± SEM (*p < 0.05 control vs. CS group and #*p* < 0.05 CS vs. AK-7 group).

The level of different cytokines was estimated in BALF. IL-5 (**Figure 5A**) level does not show any significant difference among different groups. IL-6 (**Figure 5B**) and IL-17 (**Figure 5C**) levels were significantly elevated in the CS group compared with the control and downregulated in AK-7 group in a dose-dependent manner compared with the CS-induced COPD group. Furthermore, the mRNA expression of TNF-α (**Figure 5D**) and IL-4 (**Figure 5E**) was found to be significantly increased in the CS group compared with the control group. AK-7 administration decreased the expression of IL-4 and TNF-α when compared with the CS group. The level of anti-inflammatory cytokine IL-10 (**Figure 5F**) was declined in the CS group when compared to the control, and their level was significantly elevated in AK-7 group.

### 3.5 Histopathological analysis: X-ray and staining to observe inflammation and structural alterations in the lungs

**Figure 6:**
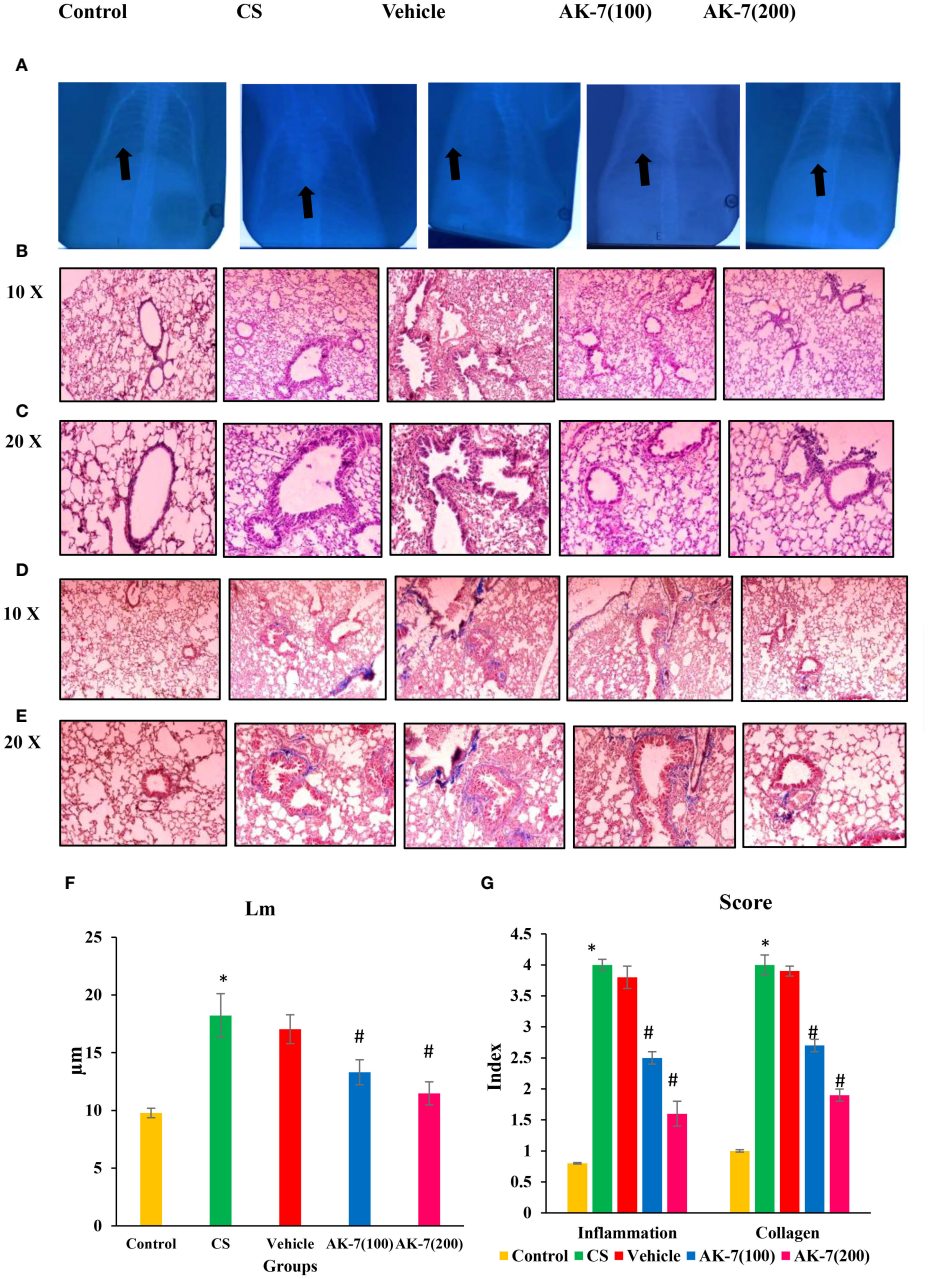
Images of mouse lungs: **(A)** X-ray analysis, **(B)** histopathological analysis of lungs (H&E) stained at ×10 **(C)** and ×20, **(D)** Masson’s trichrome (MT) staining ×10 **(E)** and ×20, **(F)** mean linear intercept (Lm), and **(G)** score of inflammation and collagen content. The control mice’s lungs show a clear area of thoracic cavity with transmission of X-rays where CS and vehicle group mice showed less transmission of X-rays. The AK-7 group shows increased transmission of X-rays across lungs in a dose-dependent manner. The control group showed regularly arranged epithelial cells, no damage in alveolar space, and no inflammation. The CS and vehicle group shows damaged alveolar space and epithelial cells along within inflammation as represented by infiltration of inflammatory cells in the peribronchiolar region. The AK-7 group dose-dependently shows ameliorated inflammation and less alveolar damage compared with the CS-exposed group. The vehicle group is showing a similar pathological condition as that found in the CS-exposed group. MT staining showed an intense blue color representing collagen in the CS group, which represents airspace remodeling in the periphery of bronchioles. The AK-7 group, in a dose-dependent manner, reduced the collagen in the peribronchiolar region. Inflammation and collagen score were significantly increased in the CS group and declined in the AK-7 group in a dose-dependent manner. Images were taken with an Olympus microscope at a magnification of ×10 and ×20. Black arrow in figure **(A)** represents area of X- ray transmission.

In clinical routine, changes in the lungs are detected using chest X-ray to observe infection, injury, and fibrosis in alveolar space and peribronchiolar areas. X-ray imaging also shows chronic diseased lung conditions, such as emphysema or fibrosis as well as complications related to these conditions. Regions with decreased X-ray transmission can be used to identify lung tissue that has been severely damaged. In normal lungs, X-rays are completely transmitted, indicating no injury and fibrosis, whereas in COPD lungs there is less transmission of X-ray across the lungs, which might be due to fibrosis or emphysema condition. The AK-7 group shows increased transmittance of X-rays compared with the COPD group, indicating the reduction of disease severity in terms of fibrosis (**Figure 6A**). However, H&E staining of the control exhibits epithelium being consistently structured, with no damage in alveolar space and no inflammatory cell infiltration. The lungs of COPD mice demonstrated loss of airway epithelial cells, inflammatory cell infiltration associated with an increase of alveolar space, and alveolar damage. In a dose-dependent manner, AK-7 administration reversed the epithelial damage, inflammation, and cellular infiltration (**Figures 6B, C**). SIRT-2 inhibition by AK-7 was successful in minimizing the structural damage resulting from CS exposure. Eventually, Lm showed significant airway enlargement in the CS-exposed group compared with the control, and AK-7 reduced the enlargement and alveolar space damage (**Figure 6F**). MT staining was performed to observe collagen deposition in airways, which represents airspace remodeling. The COPD group shows thickened bronchioles compared with the control, representing excess collagen deposition in the periphery of bronchioles. Collagen deposition was declined in AK-7 in a dose-dependent manner compared with the COPD group (**Figures 6D, E**). Inflammation and collagen content score was found to be significantly higher in the COPD group compared with the control, and this score was decreased in the AK-7 group compared with the CS-induced COPD group (**Figure 6G**).

### 3.6 SEM analysis of lung tissues

**Figure 7:**
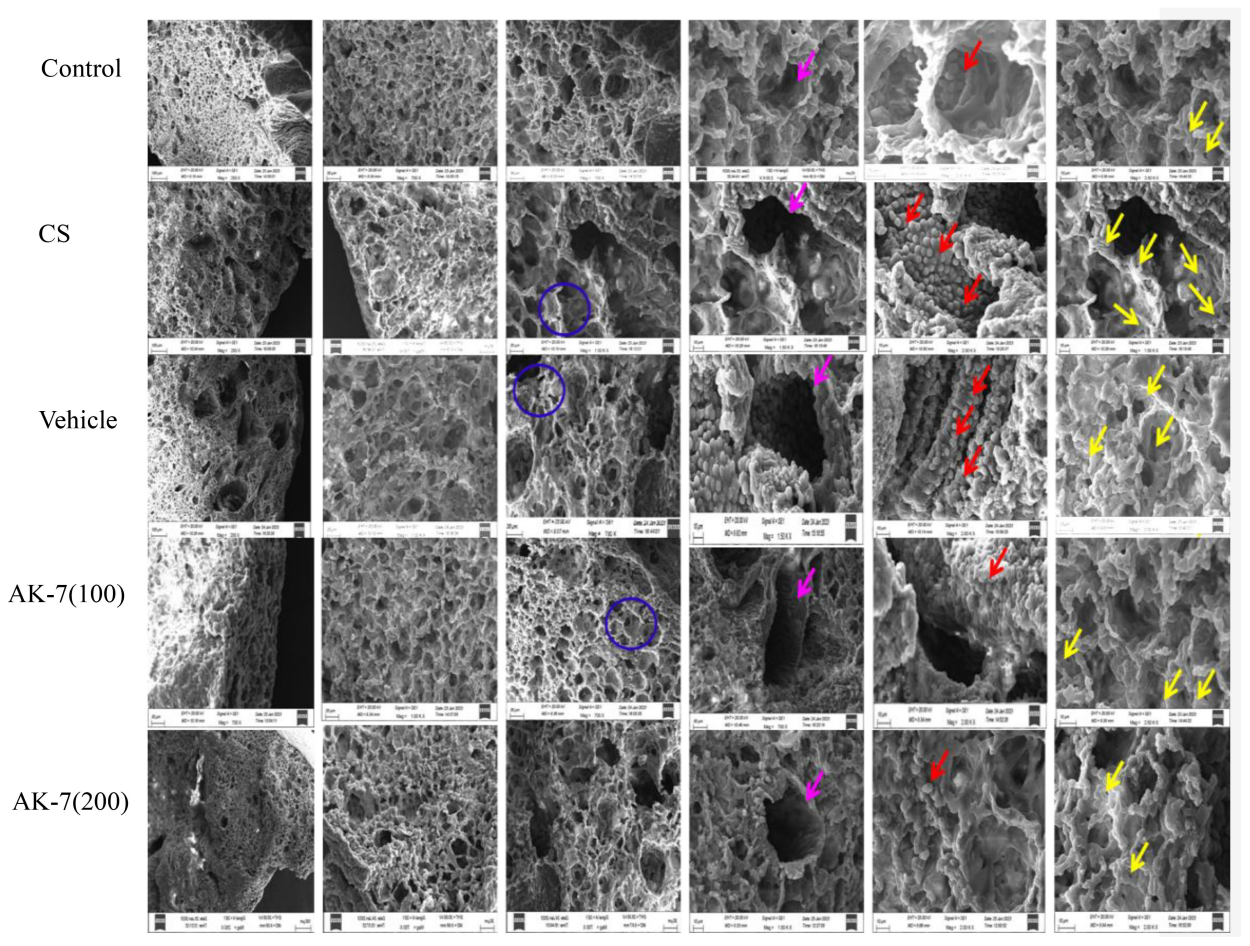
Structural analysis of the lungs through SEM. The alveolar space, respiratory tube, club cells, and alveolar septum, along with Kohn pores, were analyzed. The blue ring represents alveolar wall, the pink arrow represents respiratory tube, the red arrow marks club cells, and the yellow arrow represents Kohn pores.

SEM is a complementary and modern method of lung observation which provides a dynamic and fine view of anatomical structures and lung surfaces. The SEM analysis of control mice showed alveoli of uniform shape with oval, round, and smooth surface with a few club cells. The alveolar septum also represented a few pores as Kohn pores. The alveoli in the CS-induced COPD group were enlarged and distorted, and overlapping walls of air spaces were observed. The alveolar spaces also showed distended and destroyed respiratory bronchioles with broadened alveolar duct. The alveolar septum was observed with the presence of numerous Kohn pores of different sizes. Apart many club cells which were dome-shaped cells facing the lumen side with a smooth surface were found in the CS group. The CS group also represented emphysema, which was identified by apparent alveoli and enlarged airspaces representing airway remodeling with a widened alveolar duct and flattened transverse ridges. Such distortion was not seen in the AK-7 group representing lung protection by SIRT-2 inhibition. The alveoli of the AK-7 group represented the control lung structure of alveoli and bronchioles with a uniform shape, round or oval, and a smooth margin also. The AK-7 group showed a smaller number of Kohn pores and club cells (**Figure 7**).

### 3.7 Impact of AK-7 on the expression of p-NF-kB, p-38 MAPK, p-ERK, p-JNK, and SIRT-2

**Figure 8:**
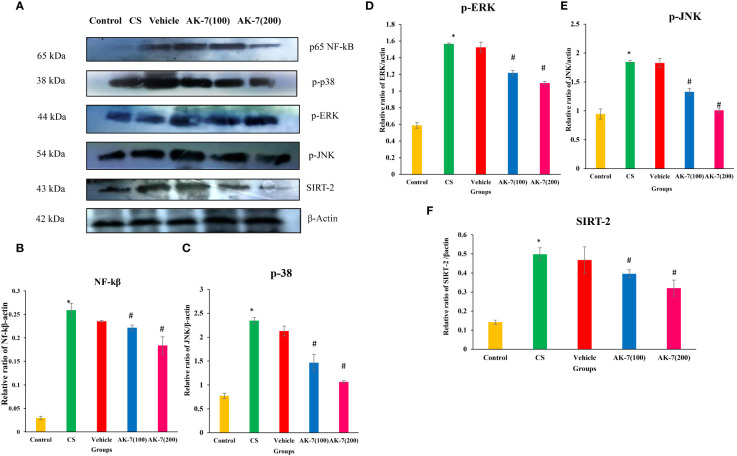
Effect of AK-7 on NF-kB, p-38, p-ERK, p-JNK, and SIRT-2 expression. **(A)** Images of the expressions of different proteins. **(B)** Protein expressions of NF-kB, **(C)** p-38, **(D)** p-ERK, **(E)** p-JNK, and **(F)** SIRT-2 against β-actin in lung tissue. The band intensities were calculated and normalized against β-actin using Image J software. The CS group showed significantly higher levels of NF-kB, p-38, p-ERK, p-JNK, and SIRT-2 expression when compared to the control group. When compared to the CS group, their expression was reduced in the AK-7 group in a dose-dependent manner. Similar to the CS-exposed group, expression was seen in the vehicle group. Number of mice per group (*n* = 8). The result is shown in mean ± SEM (*p < 0.05 control vs. CS group and #*p* < 0.05 CS vs. AK-7 group).

Western blotting was performed to evaluate the effect of AK-7 on various proteins involved in controlling inflammation through NF-kB and the MAPK signaling pathway (**Figure 8A**). The results revealed that CS exposure resulted in an elevated expression of p-NF-kB (**Figure 8B**), p-38 (**Figure 8C**), p-ERK (**Figure 8D**), p- JNK (**Figure 8E**), and SIRT-2 (**Figure 8F**) in the CS-induced COPD mice compared with the control group, and AK-7 significantly downregulated the expression of these proteins in a dose-dependent manner.

### 3.8 Assessment of iNOS and Nrf-2 gene expression through RT-PCR

**Figure 9:**
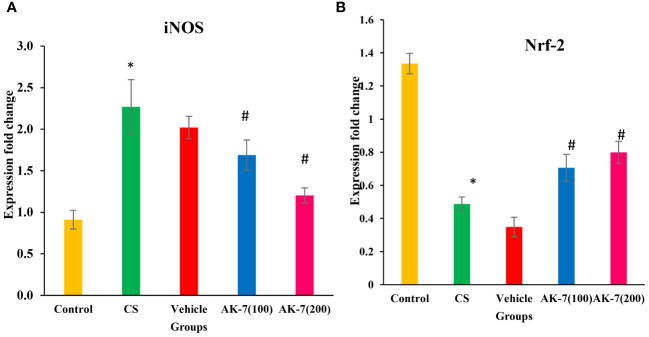
Assessment of gene expression by using specific primers. **(A)** iNOS and **(B)** Nrf-2. Gene expressions were normalized against GAPDH. The CS group shows a significant elevation in gene expression of iNOS compared with the control. The AK-7 group shows a significant reduction in the expression of this gene when compared to the CS-exposed group. Nrf-2 gene expression was significantly less in the CS group compared with the control, and its expression was enhanced in the AK-7 group compared with COPD. Number of mice per group (*n* = 8). The result is shown in mean ± SEM (*p < 0.05 control vs. CS group and #*p* < 0.05 CS vs. AK-7 group).

The gene expression of iNOS (**Figure 9A**) and Nrf-2 (**Figure 9B**) was assessed through RT-PCR. iNOS expression was significantly increased in the CS group compared with the control group. The AK-7 group showed a decrease in expression when compared to CS. However, the expression of Nrf-2 gene was significantly declined in the CS-induced COPD group compared with the control, and an increased expression was found in the AK-7 group when compared to the CS group.

### 3.9 Assessment of binding affinity of different proteins with SIRT-2

**Figure 10:**
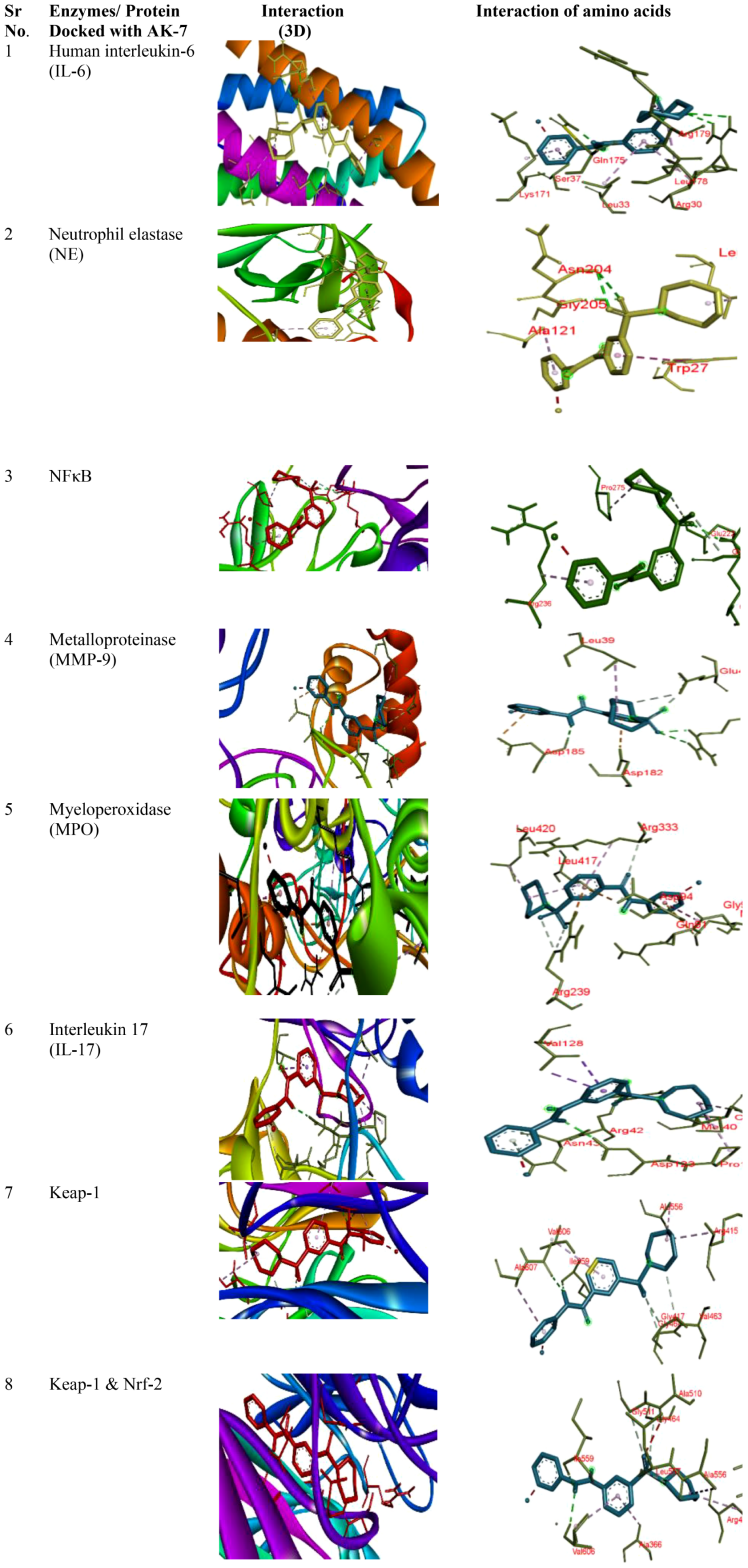
Docking of different proteins with ligand AK-7. The left panel represents the 3D interaction of the protein with the ligand, and the right panel shows the 2D docked structure.

Binding of different proteins with AK-7 was analyzed through molecular docking, and it was found that they showed a significantly good binding affinity (**Figure 10**). IL-6 shows binding energy (ΔG) -7.9 with three hydrogen bonds, and the different amino acids involved in bond formation were ARG a30, ARG a30, GLN a175, SER a37, LEU a178, ARG a179, ARG a30, LEU a33, and LYS a171. NE shows ΔG -7.8 with three hydrogen bonds, and the amino acids participating in bond formation are ASN a204, ASN a204, GLY a205, TRP a27, LEU a137, and ALA a121. NF-κB has ΔG -8.7 with three hydrogen bonds, and the amino acids in interaction were GLN a241, GLY d259, GLU a222, PRO a275, and ARG a236. Metalloproteinase (MMP9) has ΔG -8.2 with five hydrogen bonds, and amino acids in interaction were ARG a51, ARG a51, ASP a185, GLU a47, GLU a47, ASP a182, ASP a185, and LEU a39. Myeloperoxidase (MPO) has ΔG -9.9 with three hydrogen bonds, and the different amino acids involved in bond formation are ARG c239, ARG c239, ARG c333, ARG c239, ASP a94, MET a87, GLY a90, LEU c417, LEU c420, and ARG c333. IL-17 exhibits ΔG -8.1 with three hydrogen bonds, and the amino acids are ARG b42, ASP c123, ASN b43, VAL a128, VAL a128, MET b40, PRO c122, and CYS c154. Keap-1 shows ΔG -10.4 with five hydrogen bonds, and the amino acids involved in the interaction are ILE a559, VAL a606, GLY a417, GLY a464, VAL a463, ARG a415, ALA a556, VAL a606, and ALA a607. Keap-1 and Nrf-2 show a binding energy of -10.5 with five hydrogen bonds, and the amino acids involved in interactions are ILE a559, VAL a606, GLY a464, GLY a511, LEU a557, ARG a415, ALA a556, ALA a366, and VAL a606 (**Table 3**).

**Table 3:** Binding energy, number of hydrogen bonds, and amino acids involved in the interaction.

Sr. no.	Enzyme/protein	PDB ID	Ligand	Binding energy (ΔG)	No. of hydrogen bonds (drug enzyme)	Amino acids involved in the interaction
1	Human interleukin-6 (IL-6)	1ALU	AK-7	-7.9	3	ARG a30, ARG a30, GLN a175, SER a37, LEU a178, ARG a179, ARG a30, LEU a33, LYS a171
2	Neutrophil elastase (NE)	1H1B	AK-7	-7.8	3	ASN a204, ASN a204, GLY a205, TRP a27, LEU a137, ALA a121
3	NF-κB	1IKN	AK-7	-8.7	3	GLN a241, GLY d259, GLU a222, PRO a275, ARG a236
4	Metalloproteinase (MMP-9)	1L6J	AK-7	-8.2	5	ARG a51, ARG a51, ASP a185, GLU a47, GLU a47, ASP a182, ASP a185, LEU a39
5	Myeloperoxidase (MPO)	3F9P	AK-7	-9.9	3	ARG c239, ARG c239, ARG c333, ARG c239, ASP a94, MET a87, GLY a90, LEU c417, LEU c420, ARG c333
6	Interleukin 17 (IL-17)	3JVF	AK-7	-8.1	3	ARG b42, ASP c123, ASN b43, VAL a128, VAL a128, MET b40, PRO c122, CYS c154
7	Keap-1	6TYM	AK-7	-10.4	5	ILE a559, VAL a606, GLY a417, GLY a464, VAL a463, ARG a415, ALA a556, VAL a606, ALA a607
8	Keap-1 and Nrf-2	7K2F	AK-7	-10.5	5	ILE a559, VAL a606, GLY a464, GLY a511, LEU a557, ARG a415, ALA a556, ALA a366, VAL a606

## 4 Discussion

COPD is a devastating lung disease that imposes a heavy personal and social burden globally. The greatest risk factor for COPD includes exposure to harmful particles and chemicals, particularly CS. Current COPD therapy, along with smoking cessation, aims to relieve symptoms and prevent exacerbations, but disease-modifying treatment is still lacking. More progress in disease management is required to halt the alarming growth in the burden of COPD. A prolonged and inappropriate inflammatory response within the lungs is the primary pathogenic characteristic of COPD. Several researches have been conducted on the association of respiratory disease pathophysiology and SIRT regulation; however, the molecular mechanisms of SIRT-2 involved in COPD remain unknown. Hence, the present study has been aimed to examine the epigenetic modulation in COPD pathogenesis through SIRT-2 inhibition in an experimental murine model.

Ample evidence indicates that chronic exposure to CS alters the immune and inflammatory processes in the lung, causing changes in humoral and cell-mediated immune responses (47). The precise mechanism of how the innate and adaptive immune systems contribute to the development of COPD is still completely unknown. Cellular infiltration in airways is a major phenomenon and sign of inflammation, leading to the development and progression of COPD pathogenesis (48). According to recent research, a wide range of immune cell subsets is involved in the development of COPD pathophysiology, which further promotes the interplay between innate and adaptive components leading to the progression of the disease. The accumulation of macrophages and polymorphonuclear leukocytes (innate immune component) is believed for the development of the disease, whereas lymphocytes (B and T cells) as adaptive components can contribute to the progression of the disease (49). Representing the same, the present study reported airway inflammation in the CS-induced COPD group which was assessed by total and differential cell count. The increased inflammation in the airways of the COPD group corresponds to the recruitment of immune cells as observed in BALF slides which mainly included macrophages, neutrophils, and lymphocytes. On the contrary, SIRT-2 inhibition by AK-7 decreased the recruitment of cells, including macrophages, neutrophils, and lymphocytes, thereby inhibiting inflammation. The present finding was consistent with the previous finding where SIRT-2 inhibition by AGK-2 in asthmatic mice resulted in diminished cellular recruitment and SIRT-2 overexpression increased cellular recruitment to the lungs. The results of the immune cell profile further confirmed by FACS were in line with the abovementioned assertion, representing increased cell density expressing Gr-1 (neutrophils) and F4/80 (macrophages) in the CS-exposed group. On the other hand, AK-7 diminished the cell population of neutrophils and macrophages.

Moreover, lymphocytes are the cellular effectors of adaptive immunity (both B and T cells) and the distinctive hallmarks in airway inflammation, including clonal expansions and generation of immunologic memory. A better understanding of adaptive immune processes in COPD might present more effective disease interdictions, including immunoregulatory mechanisms specifying disease-associated lymphocyte subpopulations. T cells specified as CD4^+^ and CD8^+^ T lymphocytes have been recently shown to be critical for the induction of inflammation, secretion of pro-inflammatory mediators, tissue destruction, and activation of other immune cell types (B cells) in a murine model of smoke-induced emphysema (50). Eventually, CD8^+^ lymphocytes in COPD lungs are directly related to the degree of airflow limitation, but the potential contributions of CD4^+^ T-cells in the disease process also appear to be substantial. Moreover, recent studies have also reported the substantial correlation between B cell existence and COPD, where CD19 cells representing B cells delineate the severity of the disease (51). Another investigation specifies the contribution of B cells leading to emphysema phenotype in COPD. As reported, chronic inflammation in COPD corroborates the importance of T-lymphocyte responses, which was also evident in the current investigation where cell density expressing CD4^+^, CD8^+^, and CD19^+^ was increased and CD3^+^ cell population was decreased in the COPD group compared with the normal group (52). AK-7 administration modulated the cell density by decreasing CD4^+^, CD8^+^, and CD19^+^ and increasing the CD3^+^ cells. The reduction of CD3^+^ (naïve T-cell population) in different groups except the control can be attributed to their differentiation into CD4^+^ (T helper, Th) cells and CD8^+^ (T cytotoxic, Tc) cells. CD8^+^ lymphocytes appear to play a particularly important role in the development and/or progression of COPD. The present study elicited an increased CD8^+^ cell population compared with CD4+ in the COPD group, representing a major role of CD8^+^ cells in disease progression and which was in support of previous studies where CD8^+^ T cells predominated over CD4^+^ T cells in the airways and lung parenchyma of COPD patients (53). SIRT-2 inhibition by AK-7 declined the CD8^+^ and CD4^+^ cell population with respect to the COPD group. The ratio of CD4^+^/CD8^+^ less than 1 also supports the immunosuppressed physiology in COPD, which was substantially recovered to 1 by AK-7.

The two subsets of T cells (CD4^+^ and CD8^+^) further decide the fate of inflammation as Th1 (cellular immunity) and Th2 (humoral immunity). Th1 response produces interferon-gamma, IL-2, and TNF-α, which stimulate CD8^+^ T cells, NK cells, and macrophages, causing neutrophilic inflammation and potentially resulting in tissue obliteration. Th2 cells produce IL-4, IL-5, IL-10, and IL-13, which lead to increased antibody synthesis (IgE), eosinophil activation, and inhibition of macrophage activities, causing eosinophilic inflammation (54). In the present study, the cytokine profile expressing enhanced TNF-α, IL-6, and IL-17 in the COPD group and its suppression in the AK-7 group represents the Th1 paradigm response which also supports the CD8^+^ predominance over CD4^+^. The results were in support of prior findings also where early SIRT-2 inhibition evade excessive neuroinflammation by lowering the levels of IL-1, IL-6, GFAP, and TNF-α (55). Furthermore, non-statistical change in IL-5 and EPO in all groups supports non-eosinophilic condition which also sustains Th1 paradigm.

Besides Th1 and Th2 cells, Th17 also perform an important function in the pathophysiology of COPD. Th17 secretes IL-17A which causes airway epithelial and inflammatory cells to release mediators involved in inflammation. A study by Ge et al. (2020) reports the involvement of IL-17 in promoting the release of inflammatory cytokines such as IL-6 and TNF-α, which further enhances the inflammatory response (56). In the ongoing study, increased IL-17 in the COPD group and decrease in the AK-7-administered group suggest the role of IL-17 in controlling the release of IL-6 and TNF-α, thereby modulating inflammation. However, a potent anti-inflammatory cytokine (IL-10) is also very important in COPD development, as it restrains the response of Th1 cells, macrophages, and the aggregation of neutrophils. IL-10 inhibits the function of IL-17 indirectly by amplifying the inhibition of T regulatory cells (Tregs) to Th cells (57). The anti-inflammatory prospect of AK-7 might be concurrent with the increased IL-10 level, further inhibiting IL-17 synthesis by Th17 cells.

A crucial phenomenon in innate immune regulation is the recruitment of macrophages and neutrophils releasing several mediators on activation that play an important role in perpetuating chronic inflammation and emphysematous changes. Neutrophils and macrophages release a significant number of proteinases and ROS during migration, contributing to an elevated oxidative burden in smokers (58). Obligate proteolysis contributes significantly to bystander tissue damage in COPD (59). In the present study, the increased ROS and NE in the COPD group correlates with increased neutrophil recruitment in the airways, which was decreased by AK-7 administration. Furthermore, CRP and histamine signifies two other inflammatory markers of COPD. However, IL-6 and IL-17 promote CRP production, whereas IL-4 increases histamine release, suggesting that the cross-talk between IL-17 and CRP/IL-6 may further amplify the inflammatory cascade (60). The present study with increased CRP, IL-4, and histamine in the COPD group and its inhibition by AK-7 also suggests the role of Th17 in progressing COPD pathophysiology and its amelioration by AK-7.

CS-induced oxidative stress plays an important role in enhancing inflammation by regulating intracellular signaling, chromatin remodeling (histone acetylation/deacetylation), and activation of redox-sensitive transcription factors, such as NF-kB and Nrf-2 that represent a close association with inflammation, cellular growth, and stress response. Evidence suggests that Nrf-2, an important transcription factor, plays a crucial role in CS-induced COPD, where it is involved in regulating the antioxidant response and clearance of ROS and protects against oxidative stress. Upregulation of Nrf-2 improved CS-induced oxidant stress in rat lungs (61). In addition, studies also report that activation of NF-kB, a traditional transcription factor, controls the inflammatory response and is involved in cellular proliferation and infiltration and hence leads to the development of COPD. Activation of Nrf-2 regulated by redox-sensitive factors protects against CS-induced lung inflammation by regulating NF-κB (62). Studies prove that the absence of Nrf-2 can increase NF-κB activity and lead to increased cytokine production, which is related to increased oxidative stress. However, SIRT-2 deacetylates p65 in the cytoplasm, controlling the expression of particular NF-kB-dependent genes (63). Consistent with the abovementioned findings, our results also showed a significant decrease in Nrf-2 and increase in NF-κB expression in the COPD group, suggesting that inflammation is mediated through the increased expression of NF-κB. On the other hand, AK-7 increased the Nrf-2 and decreased the NF-κB gene expression, respectively, suggesting anti-inflammatory effects of AK-7 being associated with reduced NF-κB activation. Inducible iNOS is a key enzyme in the macrophage inflammatory response and the source of nitric oxide (NO) that is potently induced in response to proinflammatory stimuli by NF-κB (64). However, in the present study, it was found that the CS-induced group represented a higher iNOS expression, whereas the AK-7-administrated group effectively suppressed the iNOS level. The result might be concurrent with modulated NF-κB expression.

Studies examining the cellular and molecular mechanism of inflammation and remodeling target protease production in the lungs and airways of COPD. Exploring the molecular mechanism has shed light on the important role of kinase-based signaling cascades which are activated by environmental stimuli such as tobacco smoke and endogenous signals such as cytokines, growth factors, and inflammation-derived growth factors. It has been reported that p38MAPK contributes to airway inflammation and remodeling and regulates the contractibility and obstruction of airways by inducing the production of proinflammatory cytokines such as IL-6, TNF-α, and IL-17, thereby stabilizing the mRNAs for cytokines and chemokines (65). In the present study, the levels of phosphorylated p38, ERK, and JNK were elevated in the CS-exposed group, which were significantly declined with SIRT-2 inhibition by AK-7. MAPK are also responsible for phosphorylation at several serine and threonine residues of Nrf-2 in response to oxidative stress. Furthermore, in the ongoing study, Nrf-2 expression and kinases may be correlated, as the increased kinases might be responsible for decreased Nrf-2 expression in CS-exposed mice where, reversibly, the decreased kinases might have increased the Nrf-2 mRNA expression. Phosphorylation of Nrf-2 moderately enhances its nuclear accumulation, which further transactivates its downstream genes controlling oxidative stress.

Further lung structure as assessed by X-rays, H&E, MT, and SEM showed structural distortion in the parenchymal region, broncho-constriction, and inflammatory cell infiltration in the COPD group, and AK-7 reduces the destruction of parenchymal region and bronchioles. MT staining revealed airspace remodeling in the bronchiolar region and collagen deposition in the COPD group, which was decreased by AK-7 administration, thus inhibiting SIRT-2. The result of SEM analysis, together with X-ray analysis and light microscopy, supports the emphysema and airspace remodeling condition in the COPD group. Kohn pores are involved in aeration, and their presence in a huge number is responsible for the transfer of fluid and bacteria across the bronchiolar region, which leads to the emphysema condition in the COPD group. Club cells are specialized cells that line the airways and are essential for lung protection. These cells are mostly found in bronchioles and the basal layers of larger airways and lack cilia and the ability to produce mucus. They protect lung health by detoxifying dangerous compounds, secreting proteins that reduce inflammation, regulating immunological responses, and helping to repair damaged airways. So, the presence of numerous club cells can be attributed to the narrowing and thickening of bronchioles (66).

DMSO is an organic molecule that is widely used as a solvent in toxicology and pharmacology. It is generally accepted as nontoxic below 10% (v/v), and in practice, it is assumed that the effects of DMSO are negligible (67). In the present study, 1% DMSO is used as solvent of AK-7. The vehicle group was administered with a solvent of AK-7, and no therapeutic effect of DMSO was observed in the groups.

Apart from *in vivo* investigations, the current study also used molecular docking to provide a detail computational investigation of the AK-7 with selected inflammatory markers. An *in silico* study revealed the strong binding affinity of different inflammatory proteins such as IL-6, IL-17, MMP-9, myeloperoxidase enzyme, NE, NF-kB, Keap-1, Keap-1, and Nrf-2 with AK-7, which reflects the anti-inflammatory potency of AK-7. Furthermore, SIRT-2 modulation may regulate inflammation at a particular inflammatory site.

To summarize, in the present study, SIRT-2 has been recognized as an important factor and plays a critical role in regulating airway inflammation. SIRT-2 inhibition by AK-7 ameliorated the degree of inflammation via regulating the NF-kB and MAP kinase pathway. SIRT-2 inhibition may be used to strengthen the inflammatory mechanism in COPD.

## 5 Conclusion

The results obtained from the study of different inflammatory parameters suggest that COPD exhibits Th1 immune response, which was achieved by CD8^+^ cells and supported by Th17 cellular population. These responses are followed by the production of different cytokines, which further induce and maintain chronic inflammatory stage. These phenomena are regulated by the NF-kB and MAPK signaling pathways. Inhibition of SIRT-2 by AK-7 deacetylates NF-kB and regulates inflammatory cell infiltration, resulting in a decline in inflammatory condition.

SIRT-2 is implicated in a wide range of physiological and pathological processes, including airway diseases such as COPD. Being a trackable target, SIRT-2 inhibition in COPD by AK-7 provided a novel therapeutic candidate for preventing airway inflammation. However, certain limitations are associated with the study, where SIRT-2 knockout mice may be useful in investigating the exact mechanism of SIRT-2 in a different airway pathophysiology. This study opens up a new arena of targeting epigenetic modulators to enhance the efficiency of different drugs against COPD.

## Data availability statement

The original contributions presented in the study are included in the article/supplementary materials, further inquiries can be directed to the corresponding author/s.

## Ethics statement

The animal study was approved by Institutional Animal Ethical Committee (IAEC), Banaras Hindu University, Varanasi, India as per the letter no. BHU/DoZ/IAEC/2021-2022/017 dated 15/02/2022. The study was conducted in accordance with the local legislation and institutional requirements.

## Author contributions

VY: Conceptualization, Data curation, Investigation, Validation, Writing – original draft. VP: Formal analysis, Investigation, Validation, Writing – review & editing. PG: Software, Writing – review & editing. AS: Data curation, Investigation, Methodology, Writing – review & editing. So: Methodology, Writing – review & editing. Su: Funding acquisition, Project administration, Supervision, Writing – review & editing.
